# Advanced oxidative degradation of monoethanolamine in water using ultraviolet light and hydrogen peroxide†

**DOI:** 10.1039/d4ra05590j

**Published:** 2024-11-06

**Authors:** Atif Khan, Saima Yasin, Hamayoun Mahmood, Shabana Afzal, Tanveer Iqbal

**Affiliations:** a Department of Chemical Engineering, University of Engineering and Technology Lahore 54890 Pakistan atif.khan@uet.edu.pk; b Department of Basic Sciences and Humanities, Muhammad Nawaz Sharif University of Engineering and Technology Multan 60000 Pakistan

## Abstract

This study aims to develop a benign and commercially viable method for the degradation of monoethanolamine (MEA) in the aqueous phase *via* an ultraviolet/hydrogen peroxide (UV/H_2_O_2_) advanced oxidation process (AOP). The current investigation is novel in terms of detailed kinetic analysis and degradation mechanisms; the impact of pH and UV light intensity on MEA degradation was thoroughly examined. pH 9 was identified as the optimal condition, achieving a degradation efficiency of 76.28%, while the highest UV light intensity of 59.055 mJ cm^−2^ resulted in an 85.13% degradation efficiency. A comprehensive kinetic study highlighted the reaction rates under varying conditions, providing valuable insights and dynamics of the degradation. The mechanistic pathway of MEA breakdown, identified using Liquid Chromatography Mass Spectrometry (LCMS) analysis revealed ethylene glycol, glycolaldehyde, glycine aldehyde, glycine, carbon dioxide, and ammonium ions as the primary degradation products. These results provide both operational insights and a greater understanding of the degradation mechanism, demonstrating that UV/H_2_O_2_ AOP offers an effective and environmentally benign solution for MEA degradation. The findings make a substantial contribution to the development of MEA treatment methods that are both economically viable and sustainable.

## Abstract 2

Development of an eco-friendly and commercially feasible method for monoethanolamine (MEA) degradation in aqueous phase using ultraviolet/hydrogen peroxide (UV/H_2_O_2_) advanced oxidation process (AOP).

## Graphical abstract

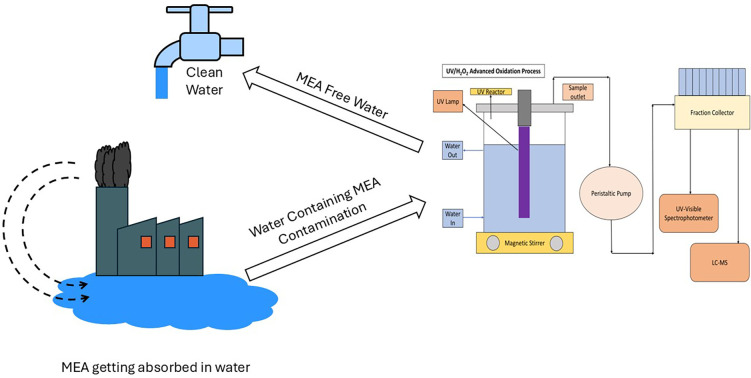

## 1. Introduction

Monoethanolamine (MEA) is an organic chemical compound from the primary alcohol group and is extensively used in various industrial applications.^1–3^ MEA is mostly employed as a gas treatment agent, specifically for the elimination of hydrogen sulfide (H_2_S) and carbon dioxide (CO_2_) from refinery streams and natural gas. In the oil and gas sector, this application is essential because it guarantees fuel purification and shields processing equipment from corrosive gases by scrubbing out sour gases. MEA is also employed in the synthesis of emulsifiers, surfactants, and other chemical products, such as agrochemicals and medicines, as an intermediate. Wastewater containing MEA is unavoidably produced as a result of its widespread use.^4^

Conventional MEA treatment methods, such as biological processes, adsorption, and membrane separation, are primarily designed to degrade the compound. However, there are some limitations associated with these processes. Biological and adsorption processes are characterized by longer degradation times and sludge formation which may pose a hazard in landfilling. Additional costs are required for sludge treatment prior to disposal. Membrane separation processes require excessive maintenance, results in further increase of operational cost. The limitations of these methods have driven researchers to seek sustainable and cost-effective alternative method. In this regard, Advanced Oxidation Processes (AOP) have emerged as a viable solution for MEA degradation by hydroxyl radical intervention. The chemical structure of the target compound and the process type significantly influence AOP performance, given the reactivity of oxidizing radicals. AOP stands out to be a promising zero-sludge process for MEA degradation, with its effectiveness depending on the rate of radical generation and their interaction with the contaminant molecules. Thus, an optimal AOP design should focus on maximizing both of these factors. Hybrid AOP systems, which operate through a similar radical-driven mechanism, have also demonstrated effective results in MEA degradation.^5^

There are several environmental and health risks associated with the release of wastewater polluted with MEA. It is well recognized that MEA, even in comparatively small amounts, is hazardous to aquatic life. Aquatic ecosystems may be disrupted as a result of its acute toxicity to fish and other aquatic life. Long-term exposure to MEA can have sub-lethal consequences in aquatic species, including altered behavior, decreased success in reproduction, and stunted growth. Due to the extensive commercial use of MEAs, wastewater from the industrial sector, particularly power plants, contains high quantities of MEAs (several hundreds of ppm). Therefore, wastewater containing MEAs must be treated in accordance with effluent discharge regulations set by the US EPA. The maximum concentration of 0.6 mg L^−1^ of MEA permitted in drinkable water is limited to 0.2 mg L^−1^ for humans, and exposure levels over 1 mg L^−1^ are considered hazardous. MEA can also cause water bodies to become eutrophic, which strains aquatic life even more by encouraging the growth of algae beyond reasonable limits and lowering dissolved oxygen levels.^4,6–8^

MEA exposure in humans might occur by several means, including as through ingestion, cutaneous contact, and inhalation. Employee exposure at work is especially important in businesses where MEA is handled or utilized frequently. Breathlessness, coughing, and respiratory discomfort might result from an acute exposure to MEA fumes. Skin irritation, redness, and dermatitis can all be consequences of dermal exposure. Long-term health impacts, including as liver and kidney damage, have been linked to chronic exposure to MEA. Wastewater containing MEAs needs to be controlled and treated in order to safeguard human and environmental health due to the possible health concerns.^4,9–14^

The present study aims to investigate the UV/H_2_O_2_ AOP for MEA degradation in aqueous solutions. Our previous work is focused on the effects of starting dosages of MEA and H_2_O_2_ on MEA degradation. The greatest degradation efficiency was determined to be 77.35% at an initial MEA dosage of 10 mg L^−1^ and H_2_O_2_ dosage of 40 mg L^−1^.^5^ Expanding these findings, the current investigation endeavors to evaluate the influence of pH and UV light intensity on the degradation efficiency of MEA, offering discernments into the optimal conditions for proficient wastewater treatment. The stability of H_2_O_2_ and the production of hydroxyl radicals are two important aspects for impact of pH on AOPs. The overall performance of the process and the rate at which degradation occurs can both be greatly impacted by changes in pH.^15–21^ Furthermore, UV light intensity is a crucial factor because it directly effects on the rate of H_2_O_2_ photolysis for ˙OH radical generation.^22–24^ To maximize MEA degradation, UV light intensity measurement and the electrical energy per reaction order (EE/*O*) calculation are essential. UV light intensity has a direct impact on photolysis rate and, in turn, on the efficiency of the advanced oxidation process. In order to maximize degrading efficiency and minimize energy consumption, UV light intensity must be properly optimized. This increases process efficiency and reduces environmental impact. We computed the UV light intensity using the electrical output of UV lamp and the entire exposed area in order to get an accurate reading.^25^ This approach is beneficial because it offers a simple way to estimate intensity without requiring complex instrumentation or analysis due to the optical features of the setup, such as reflection, refraction, or scattering effects. Using UV lamps of 4 W, 6 W, 8 W, 10 W, and 15 W electrical powers, the appropriate UV light intensities were calculated. This method makes it possible to precisely regulate and modify the UV light's intensity in order to systematically investigate how it affects MEA degradation. The current investigation attempts to determine the best conditions for optimizing MEA degradation by examining these parameters. Additionally, the study explores the mechanism of degradation and the identification of degradation products. It is crucial to understand the processes by which MEA degrades and the intermediate compounds that are produced in order to evaluate the process's completion and make sure that no hazardous byproducts are produced. Extensive analytical methods, such Liquid Chromatograph Mass Spectrometry (LCMS), make it easier to characterize these degradation products precisely.^24,26^ This work illuminates the impacts of pH and UV light intensity on the AOP of UV/H_2_O_2_, and also contributes important insights to the existing knowledge on MEA degradation. The results should help to reduce the negative effects of MEA pollution on the environment and human health by leading towards the development of more effective and efficient wastewater treatment technology.

## 2. Material and methods

MEA was obtained from Sigma Aldrich with a purity of 99% for the experimental investigation of MEA degradation. High purity of MEA is used to obtain precise and reproducible data. For oxidation purpose H_2_O_2_, purchased from Sigma Aldrich as a 30% aqueous solution. H_2_O_2_ is a main oxidizing agent responsible for generating hydroxyl radicals in aqueous medium when used in conjunction with UV light. Additionally formic acid and acetonitrile (LCMS grade) are also purchased from sigma Aldrich as elution agents for ensuring the reliability of chromatographic analysis. 0.1% solution of formic acid and pure acetonitrile is used as mobile phase and gradient phase for separation and detection of MEA and its degradation products.

### 2.1. Experimental setup

**Fig. 1:**
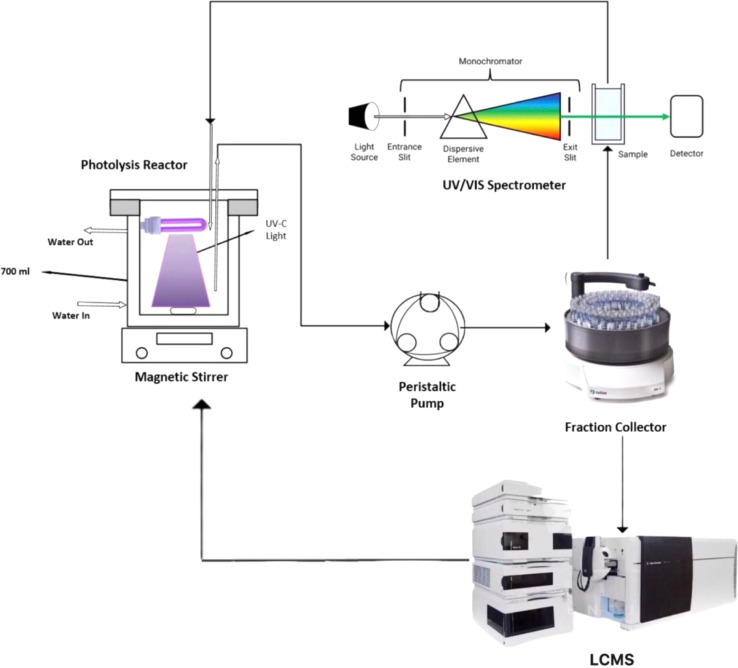
Experimental setup for MEA breakdown *via* UV/H_2_O_2_ photolysis.

A UV photolysis reactor was used to study the breakdown of MEA in water through an advanced oxidation process (Fig. 1). The influence of pH on the photolysis reactions was investigated by placing a 4 W UV-C lamp (254 nm wavelength) inside a glass reactor with a jacket. To keep the temperature steady, water was circulated around. Initially 500 ml of sample volume is added in UV reactor and after every ten minutes interval, samples (1 ml per sample) were collected from UV reactor using a fraction collector. UV lamps of different intensities (4–15 watt) for total duration of 60 minutes were placed and illuminated in the jacketed glass reactor to investigate the effect of UV light intensity on MEA degradation. The indirect iodometric approach was utilized to detect the residual H_2_O_2_ in the samples after it was quenched using sodium sulphite at a 1 : 2 molar ratio (H_2_O_2_ : sulphite). To ensure precise and reproducible results, each set of experiments was run three times over a duration of an hour.

### 2.2. Sample preparation

To examine the effects of pH and UV light intensity on MEA degradation, samples were carefully prepared. With an initial H_2_O_2_ concentration of 40 mg L^−1^, the MEA concentration was set at 10 mg L^−1^. To cover a range of values, the pH of the solution was adjusted to 3, 5, 7, 9, 10, and 12. Solutions of sodium hydroxide (NaOH) and sulfuric acid (H_2_SO_4_) were used to maintain and modify the pH levels. Several UV lamps with electrical powers of 4 W, 6 W, 8 W, 10 W, and 15 W were used to assess the impact of UV light intensity. All other parameters, such as the reaction temperature of 25 °C and the reaction duration of 60 minutes, remained constant throughout these experiments. The prepared samples were then examined to find out the MEA degradation efficiency under different pH levels and UV light intensities.

### 2.3. Intensity of UV light and electrical energy per reaction order

Determining the energy efficiency of the degradation process also requires optimizing the electrical energy per reaction order. In order to determine the most energy-efficient operating conditions, it is necessary to calculate the energy consumed per unit of MEA degraded. This calculation offers insights into the overall energy requirements. Through careful measurement and optimization of the electrical energy per reaction order as well as the UV light intensity, our goal is to improve MEA degrading efficiency while maintaining the sustainability and economic viability of the process.

Following equations are used to calculate UV light intensity and electrical energy per order of reaction.^27–29^1
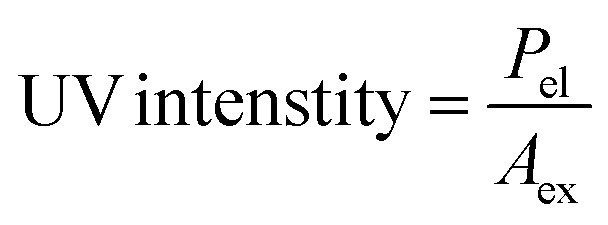
2
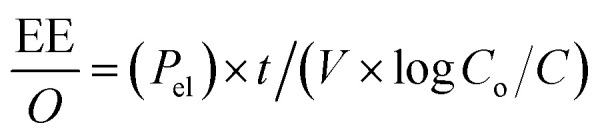
where *P*_el_ = rated power in kW, *A*_ex_ = total exposed area in cm^2^, *t* = total exposure time in min, *V* = volume of solution in liters, *C*_o_ = initial MEA concentration in mg L^−1^, *C* = final MEA concentration in mg L^−1^.

Following equation is used to find out total exposed area^5^

3 *A*_ex_ = *A*_b_ + *A*_lsa_

Here *A*_b_ = base area of solution container = π*r*^2^ and *A*_lsa_ = lateral surface area of the solution container = 2π*rh*. Here *r* is radius and *h* are height of the container in which solution is exposed to UV light. Calculations for UV light intensity and EE/*O* is provided in ESI (S1).†

### 2.4. Experimentation and analytical procedure

Using a 4 W UV lamp, the experiment started by examining the impact of pH on MEA breakdown and subsequent experiments are conducted to measure the effect of UV light intensity. Using a fraction collector, samples were frequently collected every ten minutes for subsequent analysis. A UV-visible spectrophotometer (U-1800) was used to measure the absorbance values of each sample. By calculating the absorbance values of standard MEA solutions at concentrations of 1, 2, 5, 7, and 10 mg L^−1^, a calibration curve was developed. Details for calibration curve (Fig. S1†) are provided in ESI (S1).† The concentration of MEA in the samples are determined by measuring their absorbance values and then these absorbance values are correlated with slope and *y*-intercept obtained from calibration curve to determine final MEA concentration. Liquid Chromatography-Mass Spectrometry (LCMS Agilent 1200 series) is used to analyze the samples in order to gain insight into the degradation mechanism and identify the degradation products. LCMS Column specifications, optimized LC and MS parameters (Tables S1 and S2†) and product chromatograms (Fig. S2†) along with their retention times (Table S3†) for the assessment of MEA degradation mechanisms are provided in S1.†

### 2.5. Kinetic studies and degradation efficiency calculations

Previous researchers concluded that the degradation of MEA in the aqueous phase followed a pseudo-first order reaction, which can be represented by the following equation.^30^4
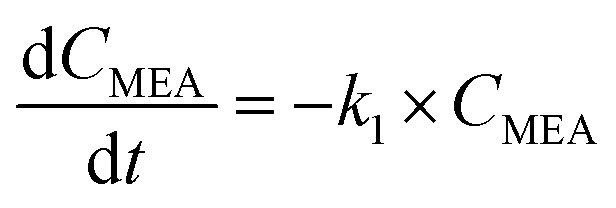
Here, *t* is the reaction time in minutes, *C*_MEA_ is the concentration of MEA in milligrams per liter, and *k*_1_ is the rate constant (min^−1^) for the first order reaction as determined by the optimal graphical data between ln(*C*/*C*_o_)_MEA_ and reaction time. It is also anticipated that the H_2_O_2_ consumption brought on by UV will follow the same pseudo-first-order reaction kinetics. H_2_O_2_ consumption rate is provided by the following formula.^30^5
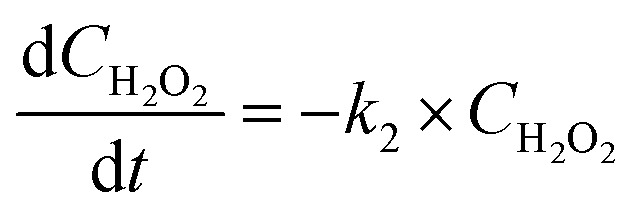
where *C*_H_2_O_2__ is the quantity of H_2_O_2_ in mg L^−1^ and *k*_2_ is the rate constant (min^−1^) determined by plotting ln(*C*/*C*_o_)_H_2_O_2__*vs.* reaction time.

The following formula is used to determine the MEA degradation efficiency (*η*%)^30^6
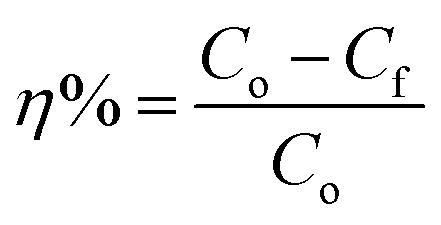
where *C*_o_ and *C*_f_ are initial and final MEA concentrations. In our previous work, at the optimum MEA and H_2_O_2_ initial dosage molar ratio value was 7.18.^5^ In the current investigation these two parameters will remain fixed to assess the impact of pH and UV light intensity.

## 3. Results and discussions

The primary objective of the study is to investigate the effects of pH and UV light intensity while holding all other variables constant in order to improve our knowledge of the kinetics of degradation reactions. Despite being an oxidant, H_2_O_2_ is unable to degrade MEA on its own and very slightly alters the concertation values following degradation. In the same way, the MEA in aqueous phase cannot be broken down by UV photolysis without the addition of H_2_O_2_. The only way to increase degradation efficiency is to use combined UV/H_2_O_2_ photodegradation.

### 3.1. Effect of pH

The current study examines the impact of various pH levels on MEA degradation kinetics. The acquired data provides detail of influence of pH on the alteration of the rate constants and final MEA concentration values.

**Fig. 2:**
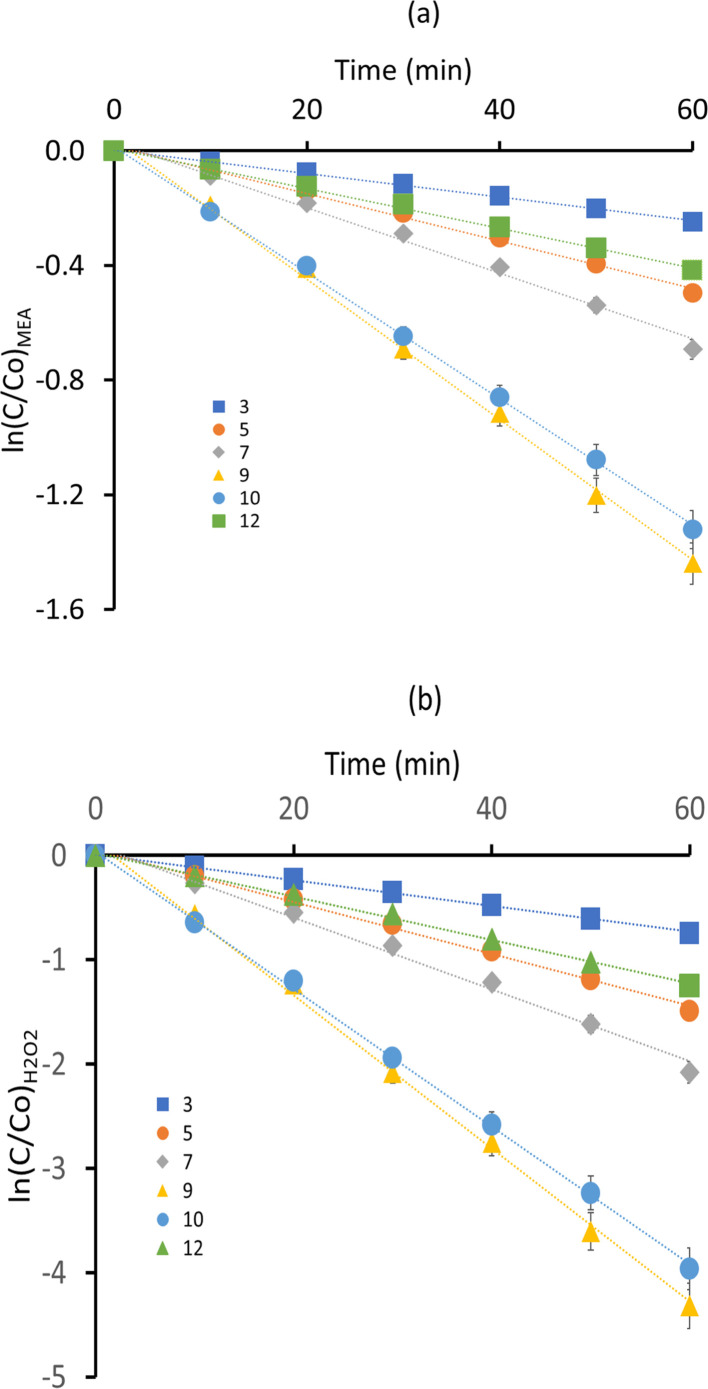
Impact of pH on MEA breakdown (a) ln(*C*/*C*_o_)_MEA_*vs.* time (b) ln(*C*/*C*_o_)_H_2_O_2__*vs.* time.

It is observed that the degradation of MEA in aqueous solution under UV/H_2_O_2_ accelerated oxidation processes followed pseudo first-order kinetics with pH values ranging from 3 to 12. The experimental data points and the fitted curve (Fig. 2a and b) closely matched, showing a persistent trend of pseudo first-order kinetics for MEA degradation in the experimental data. For MEA oxidation, hydroxyl radicals (˙OH) are needed, and their decreased availability at pH 3 indicates a slower rate of deterioration for both rate constants, *k*_1_ and *k*_2_. The degradation rate constants increased proportionately with pH values between 5 and 7, indicating that these are the more productive pH ranges for the generation of ˙OH and MEA reactivity. The optimal alkaline pH for MEA oxidation is 9, as indicated by the degradation rate constant being highest at this pH. The lesser availability of ˙OH radicals at higher alkaline pH values is likely the reason of the slight decrease in degradation rate constants at pH 10 and 12. The pseudo first-order kinetic study indicates that pH has a significant role in determining the rate of MEA degradation in UV/H_2_O_2_ processes, with pH 9 exhibiting the highest rates of degradation. These findings highlight the role that pH control plays in encouraging oxidative processes and provide useful data for enhancing MEA treatment methods in wastewater. The reaction rate constants (*k*_1_ for MEA and *k*_2_ for H_2_O_2_) related to pH variations are shown in Table 1.

**Table 1:** Rate constants *k*_1_ and *k*_2_ for different pH levels

Initial MEA dosage (mg L^−1^)	Initial H_2_O_2_ dosage (mg L^−1^)	*r*	pH	Time (min)	*k*_1_	*k*_2_	Final MEA conc. (mg L^−1^)	Residual H_2_O_2_ conc. (mg L^−1^)
10	40	7.18	3	10	0.0039301	0.01179	9.418459	35.76008
10	40	7.18	3	20			9.060036	31.83088
10	40	7.18	3	30			8.701613	28.20059
10	40	7.18	3	40			8.34319	24.85738
10	40	7.18	3	50			7.984767	21.78942
10	40	7.18	3	60			7.626344	18.98489
10	40	7.18	5	10	0.0074703	0.022411	8.97043	32.65896
10	40	7.18	5	20			8.34319	26.27598
10	40	7.18	5	30			7.71595	20.78408
10	40	7.18	5	40			7.08871	16.11625
10	40	7.18	5	50			6.46147	12.20552
10	40	7.18	5	60			5.834229	8.984895
10	40	7.18	7	10	0.0099668	0.0299	8.880824	30.81874
10	40	7.18	7	20			8.074373	23.16228
10	40	7.18	7	30			7.267921	16.89218
10	40	7.18	7	40			6.46147	11.86995
10	40	7.18	7	50			5.655018	7.957139
10	40	7.18	7	60			4.848566	5.015278
10	40	7.18	9	10	0.0222296	0.066689	8.163978	22.66094
10	40	7.18	9	20			6.551075	11.70874
10	40	7.18	9	30			4.938172	5.015
10	40	7.18	9	40			3.952509	2.571526
10	40	7.18	9	50			2.966846	1.08757
10	40	7.18	9	60			2.339606	0.533335
10	40	7.18	10	10	0.0213499	0.06405	7.895161	21.06405
10	40	7.18	10	20			6.551075	12.03363
10	40	7.18	10	30			5.117384	5.735916
10	40	7.18	10	40			4.13172	3.018921
10	40	7.18	10	50			3.325269	1.573762
10	40	7.18	10	60			2.608423	0.759614
10	40	7.18	12	10	0.006569	0.019707	9.328853	32.90621
10	40	7.18	12	20			8.791219	27.53851
10	40	7.18	12	30			8.253584	22.78877
10	40	7.18	12	40			7.626344	17.97804
10	40	7.18	12	50			7.08871	14.43759
10	40	7.18	12	60			6.551075	11.39543

**Fig. 3:**
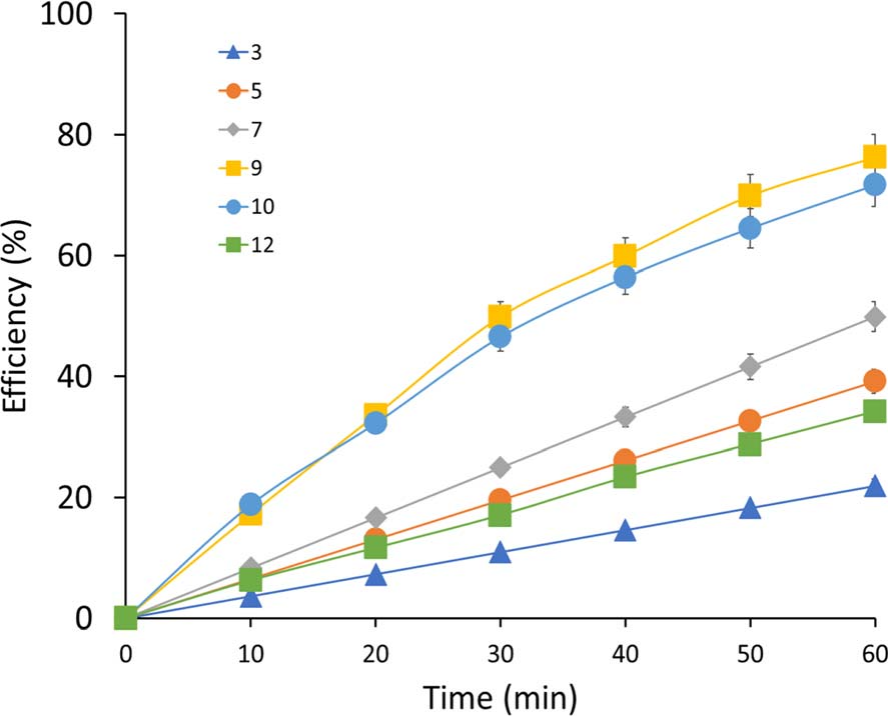
MEA degradation efficiency at different pH levels.

The effect of pH on the efficiency (Fig. 3) of the UV/H_2_O_2_ AOP for MEA degradation was investigated by measuring the pH values for 3 (21.99%), 5 (39.21%), 7 (49.84%), 9 (76.28%), 10 (71.68%), and 12 (34.20%) at 60 minutes reaction time. The results show that the degradation efficiencies of various pH values vary; pH 9 has the maximum efficiency, at 76.28%, indicating the optimal range for the oxidation of MEA and the generation of reactive oxygen species (ROS). The results indicate that MEA degradation rates are sensitive to pH changes in the UV/H_2_O_2_ process. Specifically, pH 7 demonstrated somewhat better efficiency at 49.84%, while pH 3 and pH 12 showed smaller efficiencies of 21.99% and 34.20%, respectively.

### 3.2. Effect of UV light intensity

**Fig. 4:**
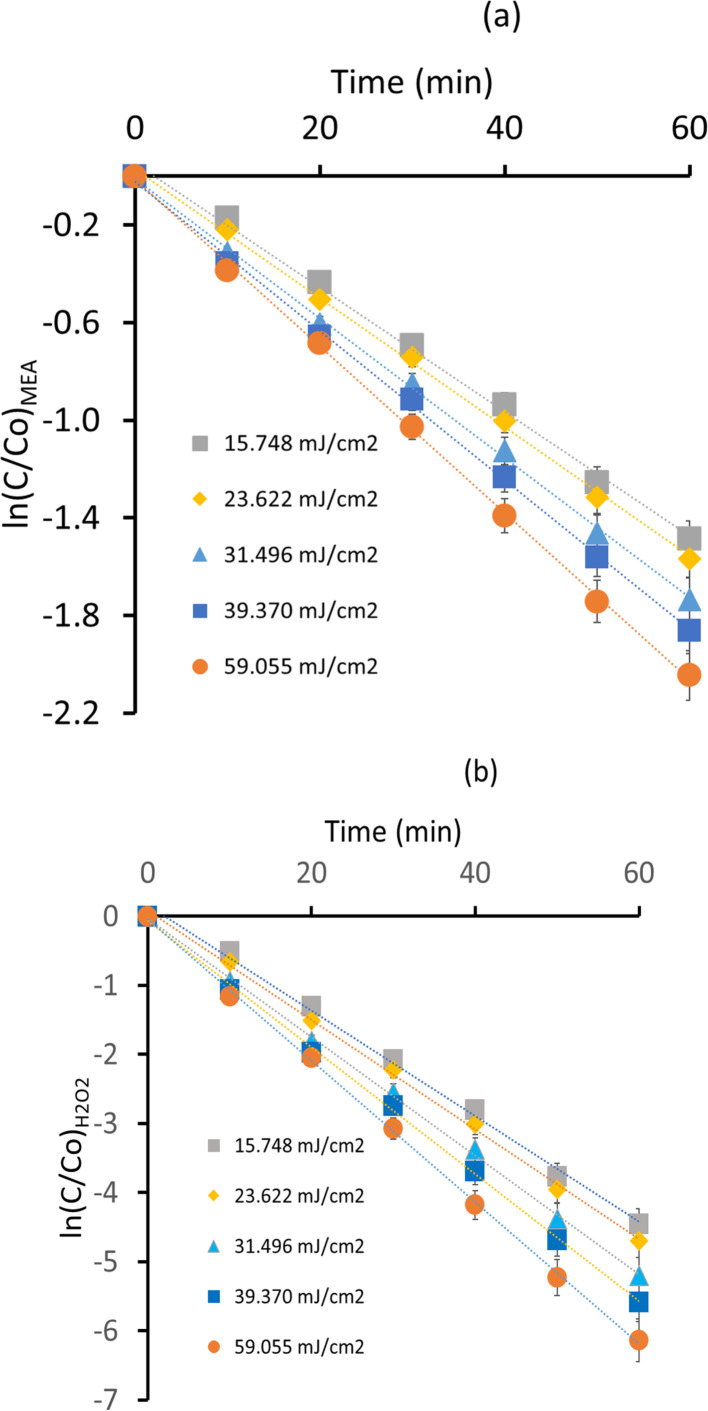
Impact of UV intensity on MEA degradation rates (a) ln(*C*/*C*_o_)_MEA_*vs.* time (b) ln(*C*/*C*_o_)_H_2_O_2__*vs.* time.

Calculations for UV light intensity and Electrical energy per reaction order are provided in ESI (S1).† By using UV/H_2_O_2_ AOP, experimental data across a range of UV intensities show that the MEA breakdown kinetics in aqueous solution exhibit a significant sensitivity to UV light intensity. Distinct UV intensity caused variations in the pseudo first order kinetics (Fig. 4a and b). The rate constants over a 60 minutes period showed a gradual but discernible degradation process at the lower UV intensity of 15.748 mJ cm^−2^. The degradation rates showed a little enhancement when the UV intensity rose to 23.622 mJ cm^−2^. As UV intensity increased more, this pattern continued. The rate constants (*k*_1_ & *k*_2_) indicated a faster breakdown of MEA at 31.496 mJ cm^−2^ than at lower intensities. The greatest UV intensities, 39.370 mJ cm^−2^ and 59.055 mJ cm^−2^, showed markedly higher rates of MEA degradation as indicated by the substantial drop in the final concentrations of MEA relative to the starting values. Values of rate constants (*k*_1_ & *k*_2_) are mentioned in Table 2.

**Table 2:** Rate constants *k*_1_ and *k*_2_ for different UV intensities

Initial MEA dosage (mg L^−1^)	Initial H_2_O_2_ dosage (mg L^−1^)	*r*	pH	UV light intensity (mJ cm^−2^)	Time (min)	*k*_1_	*k*_2_	Final MEA conc. (mg L^−1^)	Residual H_2_O_2_ conc. (mg L^−1^)
10	40	7.18	9.4	15.748	10	0.0225119	0.067536	7.71595	23.98921
10	40	7.18	9.4	15.748	20			5.923835	10.85567
10	40	7.18	9.4	15.748	30			4.579749	5.016176
10	40	7.18	9.4	15.748	40			3.594086	2.42445
10	40	7.18	9.4	15.748	50			2.608423	0.926791
10	40	7.18	9.4	15.748	60			2.070789	0.463719
10	40	7.18	9.4	23.622	10	0.024911	0.074733	7.267921	20.64909
10	40	7.18	9.4	23.622	20			5.475806	8.831094
10	40	7.18	9.4	23.622	30			4.310932	4.309072
10	40	7.18	9.4	23.622	40			3.325269	1.977655
10	40	7.18	9.4	23.622	50			2.429211	0.771021
10	40	7.18	9.4	23.622	60			1.891577	0.364034
10	40	7.18	9.4	31.496	10	0.0293365	0.088009	7.08871	15.67316
10	40	7.18	9.4	31.496	20			5.296595	6.538005
10	40	7.18	9.4	31.496	30			4.13172	3.103471
10	40	7.18	9.4	31.496	40			3.146057	1.370108
10	40	7.18	9.4	31.496	50			2.25	0.50119
10	40	7.18	9.4	31.496	60			1.712366	0.220925
10	40	7.18	9.4	39.370	10	0.0320009	0.096003	6.909498	13.73763
10	40	7.18	9.4	39.370	20			5.117384	5.581053
10	40	7.18	9.4	39.370	30			3.952509	2.571526
10	40	7.18	9.4	39.370	40			2.87724	0.991975
10	40	7.18	9.4	39.370	50			2.070789	0.369811
10	40	7.18	9.4	39.370	60			1.533154	0.150082
10	40	7.18	9.4	59.055	10	0.0351294	0.105388	6.640681	12.5342
10	40	7.18	9.4	59.055	20			4.938172	5.154156
10	40	7.18	9.4	59.055	30			3.50448	1.84217
10	40	7.18	9.4	59.055	40			2.429211	0.613557
10	40	7.18	9.4	59.055	50			1.712366	0.214906
10	40	7.18	9.4	59.055	60			1.264337	0.086506

**Fig. 5:**
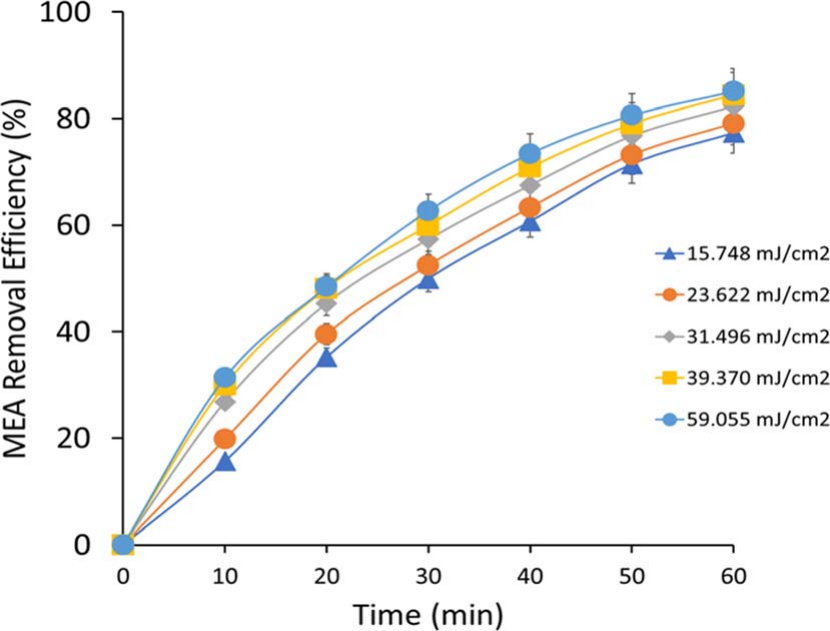
MEA removal efficiency at different UV intensities.

At a UV intensity of 15.748 mJ cm^−2^, MEA degradation efficiency (Fig. 5) was 15.67% and increased significantly throughout the course of the experiment, ending at 77.37% after 60 minutes. An increase in the rate constants for MEA degradation, which is necessary for MEA oxidation, was associated with this improvement and suggested a slow increase in the formation of ˙OH radicals. After 60 minutes, the degradation efficiency rose from 19.78% to 79.12% at a UV intensity of 23.622 mJ cm^−2^. In comparison to lower UV intensity, the related rate constants revealed quicker kinetics of MEA breakdown. This pattern demonstrated the phenomena that H_2_O_2_ absorbs more photons and produces more ˙OH radicals as a result, increasing the efficiency of MEA's removal. The degradation efficiency was initially at 26.82% at 31.496 mJ cm^−2^ UV intensity and rose rapidly to 82.32% at the end of the experiment. The related rate constants showed that the kinetics of MEA degradation were further enhanced by increased UV exposure. This study determined the optimal parameters for MEA oxidation and pollutant removal in UV/H_2_O_2_ AOP systems. The degradation efficiency rose from 29.97% to 84.46% in 60 minutes when the UV intensity was raised to 39.370 mJ cm^−2^. The related rate constants under higher photon intensity conditions indicated faster kinetics of MEA breakdown and increased generation of ˙OH radicals. This development demonstrated that changing the UV intensity can improve the efficiency of MEA removal. Ultimately, the degradation efficiency was at 31.36% and reached 85.13% at the end of the trial at a UV intensity of 59.055 mJ cm^−2^. Corresponding rate constants showed strong ˙OH radical production and fast MEA oxidation. The efficiency of MEA degradation in UV/H_2_O_2_ AOP systems is strongly impacted by increased photon flux, as demonstrated by this observation.

### 3.3. MEA degradation mechanism and pathways

**Fig. 6:**
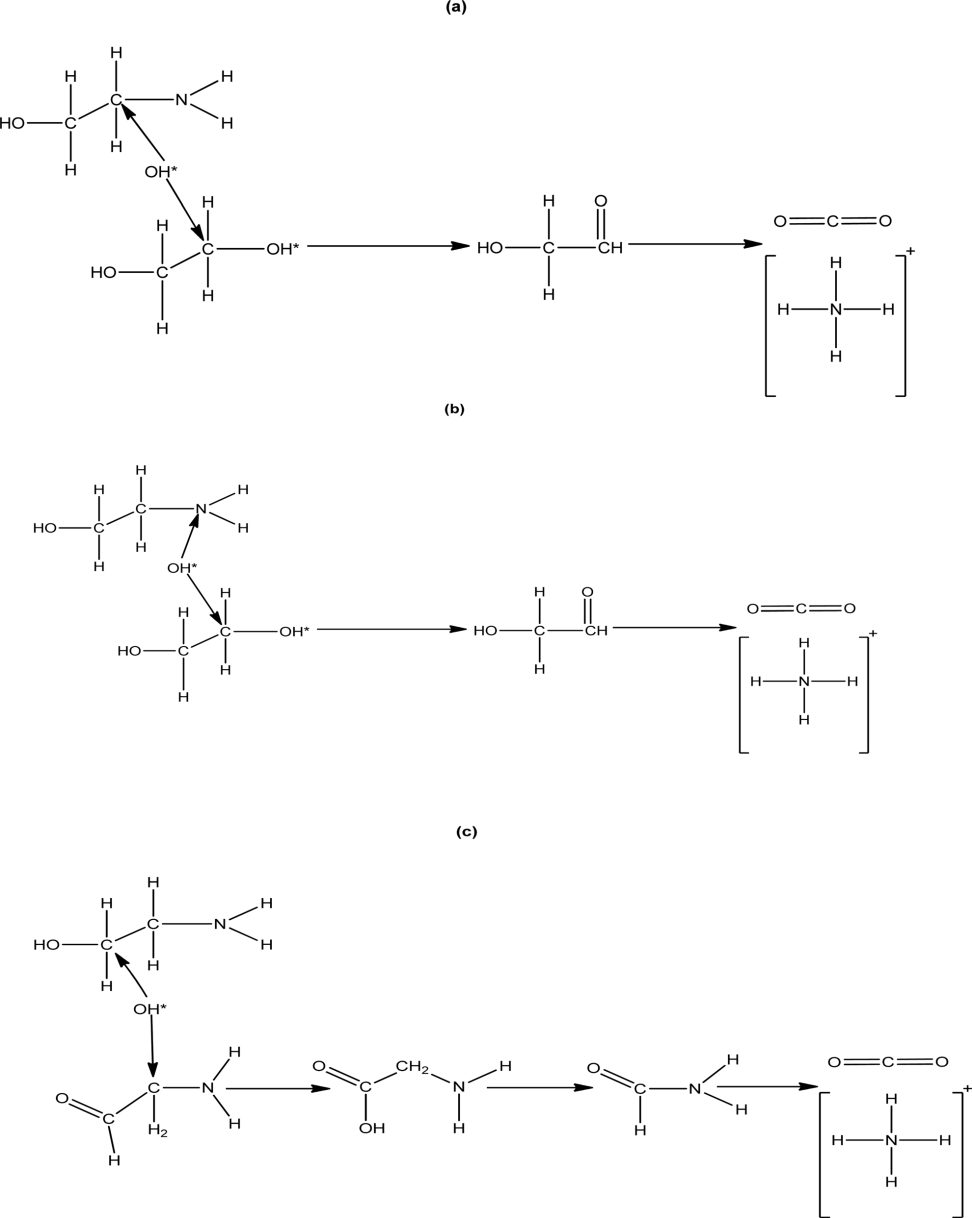
MEA degradation products (a) carbon cleavage (b) nitrogen cleavage (c) carbon cleavage.

According to the LC-MS analysis there are three pathways involved in MEA degradation. Two pathways are from carbon cleavage and one pathway from nitrogen cleavage (Fig. 6). The first carbon cleavage pathway (Fig. 6a) forms ethanolamine radical (HO–CH_2_–CH*–NH_2_) from hydroxyl radicals (˙OH) produced by H_2_O_2_ under UV light attack. After undergoing C–C bond cleavage, these radicals produce an amino radical (˙NH_3_) and ethylene glycol (OH–CH_2_–CH_2_–OH*). After that, ethylene glycol is oxidized to glycolaldehyde (HO–CH_2_–CHO), and further oxidation produced ammonia (NH_3_) and carbon dioxide (CO_2_). After that, ammonia is transformed into ammonium ions (NH_4_^+^). Degradation pathway is given below

7 OH–CH_2_–CH_2_–NH_2_ + ˙OH → OH–CH_2_–CH˙–NH_2_ + H_2_O

8 HO–CH_2_–CH˙–NH_2_ → OH–CH_2_–CH_2_–OH˙ + ˙NH_2_

9 OH–CH_2_–CH_2_–OH˙ + ˙OH → HO–CH_2_–CHO + H_2_O

10 HO–CH_2_–CHO + 2˙OH → 2CO_2_ + 3H_2_O

11
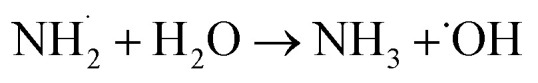

12 NH_3_ + H_2_O → NH_4_^+^ + OH^−^

Hydroxyl radicals attack MEA in the second carbon cleavage pathway (Fig. 6c), forming glycine aldehyde (NH_2_–CH_2_–CHO) and amino radical (˙NH_2_). The amino radical is subsequently converted into NH_3_ through similar degradation pathway as mentioned above. After that, glycine aldehyde is transformed into glycine (NH_2_–CH_2_–COOH), which is then further oxidized to produce ammonium ion (NH_4_^+^) and carbon dioxide (CO_2_). The degradation pathways are given below.

13 HO–CH_2_–CH_2_–NH_2_ + ˙OH → NH_2_–CH_2_–CHO˙ + ˙OH

14 NH_2_–CH_2_–CHO˙ + ˙OH → NH_2_–CH_2_–COOH

15 2NH_2_–CH_2_–COOH → 4CO_2_ + 2NH_4_^+^ + H_2_O

A MEA radical intermediate HO–CH_2_–CH˙–NH_2_ is created when hydroxyl radicals in the nitrogen cleavage pathway (Fig. 6b) remove a hydrogen atom from the nitrogen-bound carbon. After the C–N bond in this intermediate is broken, ethylene glycol and an amino radical (˙NH_3_) are produced. After that, ethylene glycol is oxidized to glycolaldehyde (HO–CH_3_–CHO), which is oxidized once more to produce ammonia (NH_3_) and carbon dioxide (CO_2_). After that, ammonia is transformed into ammonium ions (NH_4_^+^):

16 HO–CH_2_–CH_2_–NH_2_ + ˙OH → HO–CH_2_–CH˙–NH_2_ + H_2_O

17
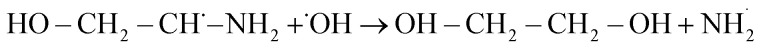

18 OH–CH_2_–CH_2_–OH + ˙OH → HO–CH_2_–CHO + H_2_O

19 HO–CH_2_–CHO + 2˙OH → 2CO_2_ + 3H_2_O

20
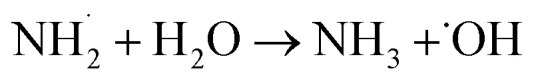

21 NH_3_ + H_2_O → NH_4_ + OH^−^

These pathways showed that the way MEA is changed under oxidative conditions, resulting in the ultimate products of ethylene glycol, glycolaldehyde, glycine aldehyde, glycine, carbon dioxide (CO_2_), and ammonium ion (NH_4_^+^). The detection of CO_2_ and NH_4_^+^ in samples are detected by titration method. Details of the method applied is provided in S1.† Understanding these mechanisms is crucial in order to optimize the AOP and regulate the formation of particular intermediates during MEA breakdown.

## 4. Conclusions and recommendations

The study is aimed to optimize pH and UV light intensity in order to improve the MEA breakdown *via* UV/H_2_O_2_ AOP. A pH range of 3 to 12 was examined in the study; pH values of 3, 5, 7, 9, 10, and 12 were taken into consideration. The results showed that at a pH of 9, the greatest degradation of MEA was attained, indicating that this pH level greatly increases the degradation efficiency. However, on the basis of our previous work it was found that 9.4 is the optimal pH for maximum MEA degradation since it greatly increases the process's efficiency. The study also showed that the degradation rate of MEA is positively impacted by increasing UV light intensity from 15.478 to 59.055 mJ cm^−2^. In particular, 325 kW h m^−3^ at 15.748 mJ cm^−2^, 340 kW h m^−3^ at 23.622 mJ cm^−2^, 375 kW h m^−3^ at 31.496 mJ cm^−2^, 405 kW h m^−3^at 39.370 mJ cm^−2^, and 440 kW h m^−3^ at 59.055 mJ cm^−2^ were determined to be the energy requirements for various UV intensities. These figures highlight the point that energy demand rises with increasing UV light intensities, which is important information to consider for assessing cost effectiveness of the process. The breakdown products that were found were ammonium ions, carbon dioxide, ethylene glycol, glycolaldehyde, and glycine. Even with the encouraging outcomes, it is still difficult to achieve a degradation efficiency of more than 90%. Future research should investigate the application of catalysts to further improve the efficiency of degradation. Experiments involving higher UV light intensity and broader pH variation could reveal additional insights. Though effective and generally safe, optimizing these aspects will be key to develop a more efficient, benign and economically viable treatment method for commercial-scale applications.

## Data availability

The data that support the findings of this study are available from the corresponding author upon reasonable request.

## Conflicts of interest

The authors state that none of the work described in this study would have been impacted by any known competing financial interests or personal relationships.

## Supplementary Material

RA-014-D4RA05590J-s001
