# Supplementary material for: Advanced oxidative degradation of monoethanolamine in water using ultraviolet light and hydrogen peroxide
Source: RSC Adv. 2024 Nov 6;14(45):33223–32. doi: 10.1039/d4ra05590j (PMC11539933; doi:10.1039/d4ra05590j)
Supplement: RA-014-D4RA05590J-s001 [file RA-014-D4RA05590J-s001.pdf]

Supplementary Information (SI) for RSC Advances.
This journal is © The Royal Society of Chemistry 2024

Calibration Data for calculating MEA Concentration:

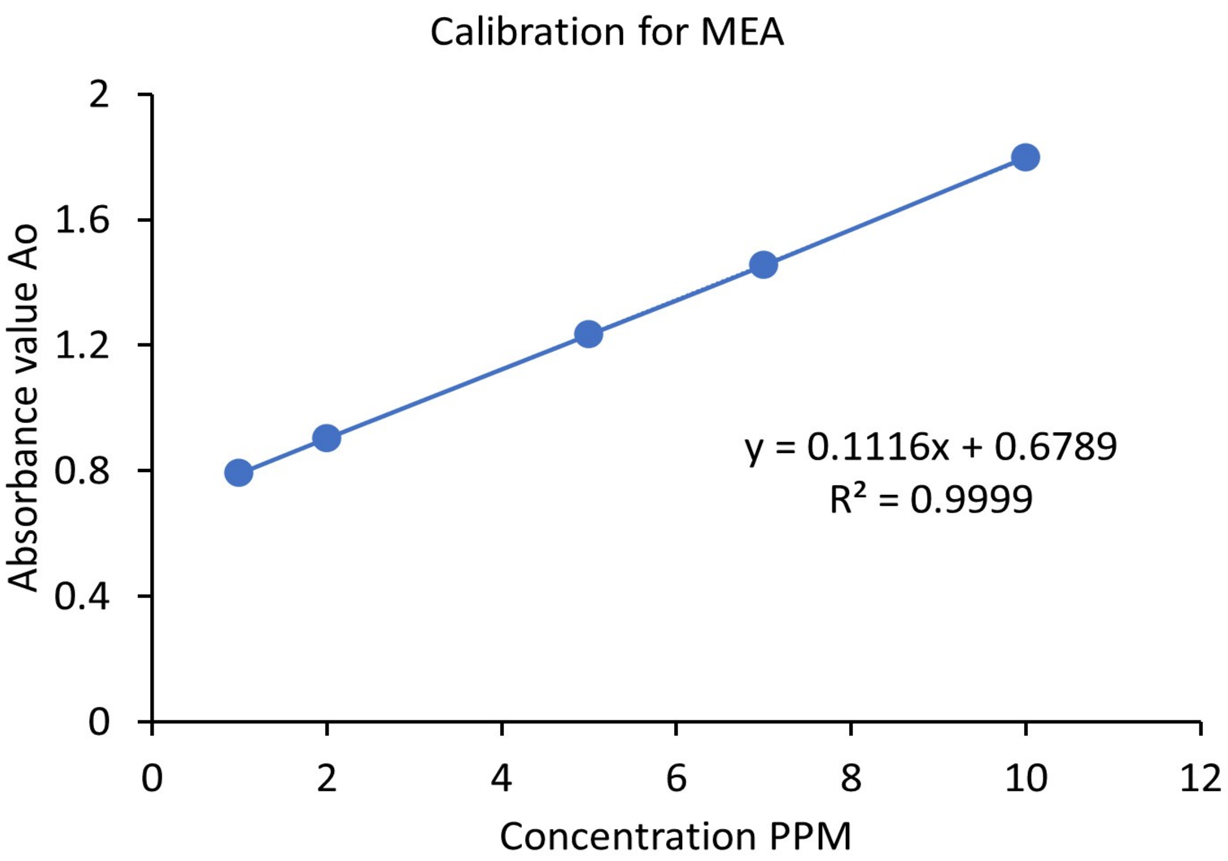

Figure S1: Calibration curve generated from UV-Visible Spectrophotometer for calculation of MEA Concentration

Chemicals and reagents:

MEA, with a minimum purity of 99%, was purchased from Sigma Aldrich for use in the experimental investigation. H_2O_2 obtained as a 30% solution, was sourced from Sigma Aldrich and utilized as an oxidant in the advanced oxidation process. Formic acid and Acetonitrile for elution solvents (LCMS grade) are also purchased from Sigma Aldrich.

Optimized Parameters for LCMS:

For MEA degradation mechanisms and products following are the optimized parameters for LC (liquid chromatography) and MS (mass spectrometer)

Column Specifications:

Chromatographic separation is achieved using a reversed-phase C-18 column (YMC-Triart, 150 x 2.0 mm, 5 µm particle size). The software used for data analysis was Agilent Mass Hunter. Electrospray Ionization (ESI) mode of ionization was employed.

Table S1: Optimized parameters of LC

Eluent	0.1 % formic acid, acetonitrile	
Mobile phase gradient	Time (min)	Mobile phase ratio (% Acetonitrile)
	0	10
	0.8	10
	1.5	90
	3	90
	4	10
	10	10
Flow rate (ml/min)	0.5	
Injection volume (µL)	30	
Column temperature (^{o}C)	30	

Table S2: Optimized parameters for MS

Parameter	Value
Drying gas temperature (^oC)	300
Nebulizer pressure (psi)	15
Gas flow (Liters/min)	6
Capillary voltage (V)	4000

Table S3: MEA Degradation products and their retention times

MEA	Degradation Products	Retention Time
$OH-CH_2-CH_2-NH_2$	Ethylene Glycol ($OH-CH_2-CH_2-OH$*)	0.62
	Glyco-aldehyde ($HO-CH_2-CHO$)	0.97
	Glycine aldehyde (NH_2-CH_2-CHO)	1.23
	Glycine (NH_2-CH_2-COOH)	1.78

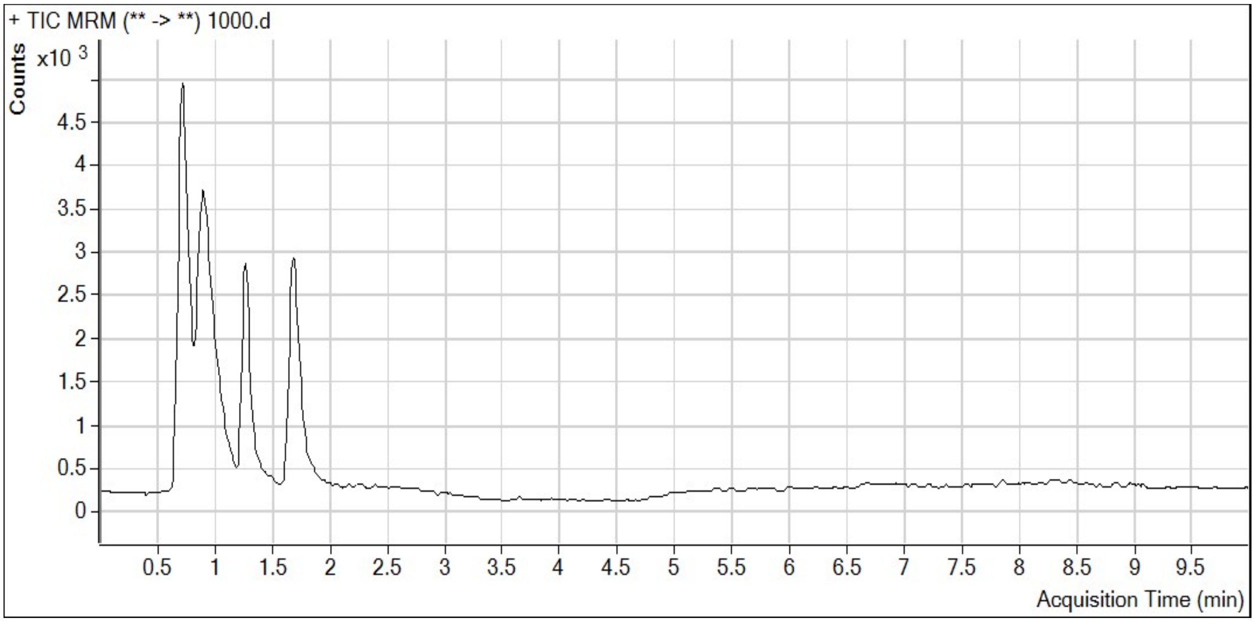

Figure S2: LCMS Chromatograms for MEA Degradation Products

Carbon dioxide (CO_2) and Ammonium ion (NH_4^+) are detected in aqueous samples by titration method

Procedure for CO_2 detection:

- The CO_2 in the aqueous sample is absorbed using sodium hydroxide (NaOH). When CO_2 and NaOH reacted, sodium bicarbonate is formed
- Pass the sample through an absorption solution of sodium hydroxide (NaOH) to capture CO_2.
- To the mixture, a few drops of phenolphthalein are added. When phenolphthalein is dissolved in basic solutions, it turns pink and turns colorless when neutralized with acid.
- A standard hydrochloric acid (HCl) solution is used to titrate the above solution.
- Following reaction takes place during titration

$$NaHCO_3 + HCl \rightarrow NaCl + CO_2 + H_2O$$

Procedure for Ammonium ion (NH_4^+) detection

- Dilute hydrochloric acid (HCl) 0.1 M solution is used to absorb the solution resulting in the formation of NH_4Cl
- Sodium hydroxide (NaOH) is used, to titrate the above solution.
- Before titration Methyl orange is added in the solution
- Titration is continued until the color of the solution is changed
- Following reaction takes place during titration

$$NH_4Cl + NaOH \rightarrow NH_3 + H_2O + NaCl$$

Calculation of UV light intensities and electrical energy per order of reaction:

UV light intensity and Electrical energy per order (EE/O) values are calculated by using the equations mentioned in section 2.3. The UV light intensities and EE/O are calculated at maximum degradation efficiency which was 77.34% at room temperature, pH = 9.4 and total reaction time = 60 minutes in our previous work[1]. The measured initial and final concentration values at these conditions were 9.10 mg/L and 2.06 mg/L.

UV Intensity and electrical energy per order for 4W UV light:

UV light intensity

Area of the base of solution container (A_b) = 44.1964 cm^2 (r = 3.75 cm, π= 3.142)
Lateral surface area of solution container (A_{LSA}) = 209.785 cm^2 (r = 3.75 cm, π= 3.142, h = 8.9cm)
Total exposed area of UV light (A_{ex}) = 254 cm^2
UV light Intensity = (4/254) *1000 = 15.748 mJ/cm^2

EE/O at highest removal efficiency

Reaction order (n) = 1, Rated Power of UV lamp (P_{el}) = 0.004KW, Volume the solution (V) = 0.5L, exposure time (t) = 60 min and log (Co/C) = 0.65
EE/O = 0.004*60*/ (0.5*0.65) = 0.325 kWh/L = 325 kWh/m^3

UV Intensity and electrical energy per order for 6W UV light:

UV light intensity

Area of the base of solution container (A_b) = 44.1964 cm^2 (r = 3.75 cm, π= 3.142)
Lateral surface area of solution container (A_{LSA}) = 209.785 cm^2 (r = 3.75 cm, π= 3.142, h = 8.9cm)
Total exposed area of UV light (A_{ex}) = 254 cm^2
UV light Intensity = (6/254) *1000 = 23.622 mJ/cm^2

EE/O at highest removal efficiency

Reaction order (n) = 1, Rated Power of UV lamp (P_{el}) = 0.006KW, Volume the solution (V) =

0.5L, exposure time (t) = 60 min and log(Co/C) = 0.68
EE/O = 0.006*60/(0.5*0.68) = 0.34 kWh/L = 340 kWh/m^3

UV Intensity and electrical energy per order for 8W UV light:

UV light intensity

Area of the base of solution container (A_b) = 44.1964 cm^2 (r = 3.75 cm, π= 3.142)
Lateral surface area of solution container (A_{LSA}) = 209.785 cm^2 (r = 3.75 cm, π= 3.142, h = 8.9cm)
Total exposed area of UV light (A_{ex}) = 254 cm^2
UV light Intensity = (8/254) *1000 = 31.496 mJ/cm^2

EE/O at highest removal efficiency

Reaction order (n) = 1, Rated Power of UV lamp (P_{el}) = 0.008KW, Volume the solution (V) = 0.5L, exposure time (t) = 60 min and log(Co/C) = 0.75
EE/O = 0.008*60/(0.5*0.75) = 0.375 kWh/L = 375 kWh/m^3

UV Intensity and electrical energy per order for 10W UV light:

UV light intensity

Area of the base of solution container (A_b) = 44.1964 cm^2 (r = 3.75 cm, π= 3.142)
Lateral surface area of solution container (A_{LSA}) = 209.785 cm^2 (r = 3.75 cm, π= 3.142, h = 8.9cm)
Total exposed area of UV light (A_{ex}) = 254 cm^2
UV light Intensity = (10/254) *1000 = 39.370 mJ/cm^2

EE/O at highest removal efficiency

Reaction order (n) = 1, Rated Power of UV lamp (P_{el}) = 0.008KW, Volume the solution (V) = 0.5L, exposure time (t) = 60 min and log(Co/C) = 0.81
EE/O = 0.01*60*/(0.5*0.81) = 0.405 kWh/L = 405 kWh/m^3

UV Intensity and electrical energy per order for 15W UV light:

UV light intensity

Area of the base of solution container (A_b) = 44.1964 cm^2 (r = 3.75 cm, π= 3.142)
Lateral surface area of solution container (A_{LSA}) = 209.785 cm^2 (r = 3.75 cm, π= 3.142, h = 8.9cm)
Total exposed area of UV light (A_{ex}) = 254 cm^2
UV light Intensity = (15/254) *1000 = 59.055 mJ/cm^2

EE/O at highest removal efficiency

Reaction order (n) = 1, Rated Power of UV lamp (P_{el}) = 0.008KW, Volume the solution (V) = 0.5L, exposure time (t) = 60 min and log(Co/C) = 0.88
EE/O = 0.015*60*/(0.5*0.88) = 0.44 kWh/L = 440 kWh/m^3

References:

1. Khan, A., et al., *Mono-ethanolamine breakdown by UV/hydrogen peroxide via MEA photolysis: kinetics, energy rate/order and degradation efficiency for mono-ethanolamine wastewater treatment.* Brazilian Journal of Chemical Engineering, 2024: p. 1-12.
